# Maternal Weight Predicts Children's Psychosocial Development via Parenting Stress and Emotional Availability

**DOI:** 10.3389/fpsyg.2016.01156

**Published:** 2016-08-10

**Authors:** Sarah Bergmann, Andrea Schlesier-Michel, Verena Wendt, Matthias Grube, Anja Keitel-Korndörfer, Ruth Gausche, Kai von Klitzing, Annette M. Klein

**Affiliations:** ^1^IFB AdiposityDiseases, Leipzig University Medical CenterLeipzig, Germany; ^2^Department of Child and Adolescent Psychiatry, Psychotherapy and Psychosomatics, University of LeipzigLeipzig, Germany; ^3^Department of Developmental Psychology, Friedrich-Schiller-University of JenaJena, Germany; ^4^CrescNet gGmbH, University of LeipzigLeipzig, Germany

**Keywords:** obesity, parenting stress, emotional availability, psychosocial development, weight development

## Abstract

**Introduction:** Maternal obesity has been shown to be a risk factor for obesity in children and may also affect children's psychosocial outcomes. It is not yet clear whether there are also psycho-emotional mechanisms explaining the effects of maternal weight on young children's weight and psychosocial development. We aimed to evaluate whether maternal body mass index (BMI), mother–child emotional availability (EA), and maternal parenting stress are associated with children's weight and psychosocial development (i.e., internalizing/externalizing symptoms and social competence) and whether these predictors interact with each other.

**Methods:** This longitudinal study included three assessment points (~11 months apart). The baseline sample consisted of *N* = 194 mothers and their children aged 5–47 months (*M* = 28.18, *SD* = 8.44, 99 girls). At *t*_1_, we measured maternal weight and height to calculate maternal BMI. We videotaped mother–child interactions, coding them with the EA Scales (fourth edition). We assessed maternal parenting stress with the Parenting Stress Index (PSI) short form. At *t*_1_ to *t*_3_, we measured height and weight of children and calculated BMI–SDS scores. Children's externalizing and internalizing problems (*t*_1_–*t*_3_) and social competence (*t*_3_, *N* = 118) were assessed using questionnaires: Child Behavior Checklist (CBCL 1.5–5), Strengths and Difficulties Questionnaire (SDQ: prosocial behavior), and a checklist for behavioral problems at preschool age (VBV 3–6: social-emotional competence).

**Results:** By applying structural equation modeling (SEM) and a latent regression analysis, we found maternal BMI to predict higher BMI–SDS and a poorer psychosocial development (higher externalizing symptoms, lower social competence) in children. Higher parenting stress predicted higher levels of externalizing and internalizing symptoms and lower social competence. Better maternal EA was associated with higher social competence. We found parenting stress to serve as a mediator in the association between maternal weight and children's psychosocial outcomes. Moreover, children of mothers with an elevated BMI were at greater risk of lower social competence only when their mothers showed low levels of maternal EA (moderation).

**Conclusion:** Interventional studies are needed that investigate the causal pathways between parenting stress, mother–child interaction quality and child outcomes. These aspects might be targets to improve the psychosocial development of the offspring of overweight or obese mothers.

## Introduction

In developed and developing countries, the prevalence rates for overweight and obesity in adults have increased substantially over the past three decades (Ng et al., 2014). In the light of this development, the health care system faces an enormous clinical and economic burden as obesity increases the risk for negative health consequences, e.g., cardiovascular disease, type 2 diabetes, several cancers, and a diminished life expectancy (Haslam and James, 2005). Although the rate of increase has decreased in the last 10 years, especially in developed countries, prevalence numbers are still high. Compared to 36.9% in men, the global proportion of overweight in women is 38% (Ng et al., 2014), and affects women of childbearing age as well. Once obese women become mothers, maternal obesity may represent a health risk not only for themselves, but also for their offspring.

In our longitudinal study we aimed to evaluate possible consequences of maternal obesity for young children's weight and psychosocial development. We further investigated whether parenting stress and the quality of the mother–child interaction (indicated by emotional availabilty; Biringen and Easterbrooks, 2012) play crucial roles in the association of maternal obesity and children's weight and psychosocial development.

A large volume of research demonstrates that parental overweight or obesity is an important risk factor for the development of childhood overweight and obesity and for an increased body fat mass in children (Whitaker, 2004; Reilly et al., 2005; Linabery et al., 2013; Gaillard et al., 2014). The factors behind the transmission of overweight and obesity from parents to their children are assumed to be genetic, epigenetic, and environmental, and seem to be strongly interwoven. As most of the genetic variability in the body mass index (BMI) is still unexplained (Locke et al., 2015) and as prevalence rates for obesity have risen too extensively and too rapidly over recent years for genetic variation to provide a full explanation of the obesity risk (Rosenbaum and Leibel, 1998; Waterland, 2008), there is room for alternative explanations such as environmental influences. Furthermore, stronger associations of maternal BMI than paternal BMI with child BMI and other child adiposity outcomes have been reported in several studies (e.g., Lawlor et al., 2007; Linabery et al., 2013; Gaillard et al., 2014). On the one hand, these results emphasize the need to take into account direct intrauterine effects, such as proposed by the “fetal overnutrition hypothesis” (Lawlor et al., 2007), which assumes that permanent changes in energy metabolism, neuroendocrine system functioning, and appetite control of the fetus are caused by prenatal influences associated with higher maternal BMI (e.g., high plasma concentrations of glucose or free fatty acids). On the other hand, these results also stress the necessity to consider the postnatal environment shared with or provided by mothers (Linabery et al., 2013; Gaillard et al., 2014).

Particularly during the early childhood years, in most families mothers are the primary caregivers of their children (Yeung et al., 2001) and shape children's eating habits through feeding and parenting practices (Birch and Ventura, 2009). By providing adequate responsiveness, attunement and emotional accessibility within the mother–child relationship, mothers can promote the development of healthy emotion regulation in their children (Easterbrooks et al., 2000, 2012), and this is associated with a healthier response to stress (Little and Carter, 2005; Kertes et al., 2009; Pillai Riddell et al., 2011; Atkinson et al., 2015).

Stress and maladaptive emotion regulation strategies lead to an increased intake of (comfort) food (Oliver et al., 2000; Zellner et al., 2006; Evers et al., 2010). As adaptive emotion regulation strategies appear to protect from overweight and obesity (Schlam et al., 2013), the mother–child relationship as a source of healthy emotion regulation has increasingly been considered in studies of obesity development. Some studies showed that children who were exposed to low maternal sensitivity (Rhee et al., 2006; Wu et al., 2011; Anderson et al., 2012) and showed insecure attachment patterns (Anderson and Whitaker, 2011; Anderson et al., 2012) were at greater risk of overweight and obesity. Also, insecure parental attachment patterns were associated with children's overweight and obesity (Mazzeschi et al., 2014). Hence, it can be assumed that the quality of the mother–child relationship may be an additional factor that influences children's weight development, and might partially mediate the link between maternal weight and children's weight. Alternatively, an emotionally well-balanced mother–child interaction might be a (moderating) factor that protects against childhood overweight or obesity. To our knowledge, this kind of interaction between biological and psychosocial factors has not been explicitly investigated so far.

Among the multiple characteristics of the environment shared with or provided by mothers, another possibly confounding environmental factor—parental stress—has to be considered as a potential additional risk factor promoting the development of obesity in children. Parental stress may directly affect children's risk of becoming obese by fostering stress and emotional arousal in the children themselves. This may lead to an increase in the secretion of stress hormones, which in turn can lead to a greater accumulation of fat in visceral depots (Anagnostis et al., 2009). Furthermore, stress or emotional arousal in children could also promote the consumption of sweet foods and eating in the absence of hunger as a comfort mechanism (Michels et al., 2015) as well as lower levels of physical activity (Roemmich et al., 2003). Parental stress may also indirectly affect children's risk of becoming obese due to its adverse influence on health socialization behaviors provided by parents, e.g., greater parental reliance on fast food, lower parental monitoring of children's sedentary behavior (Parks et al., 2012), or an uninvolved parental feeding style (Hughes et al., 2015).

Support for these assumptions is provided by a recent meta-analysis across longitudinal and cross-sectional studies (Tate et al., 2015) documenting a greater risk of childhood obesity when mothers experienced stress (e.g., financial strain, serious life events, parental worries etc.). For obese mothers, stigmatization by strangers and family members has to be considered as an important source of stress (Vartanian et al., 2014) which may affect their children as well. Stressors perceived by mothers to arise uniquely from the caregiving role, however, may affect children even more directly (Abidin, 1992, 1995). Some studies (Stenhammar et al., 2010), although not all (e.g., Koch et al., 2008; Moens et al., 2009), found maternal parenting stress to be linked to overweight in children. Furthermore, women with a greater BMI in particular seem to experience greater stress (Liu and Umberson, 2015). This leads to the conclusion that maternal parenting stress may represent an additional (moderating) risk factor for the development of overweight or obesity in the child, or may at least partially mediate the relationship between maternal weight and children's weight development.

Apart from being linked to adverse physical conditions in the child, some studies also suggest a potential impact of maternal obesity on children's mental health. In two reviews based on a total of 23 studies (Van Lieshout et al., 2011; Van Lieshout, 2013), 18 studies supported the conclusion that maternal obesity (prior to or during gestation) is linked to neurodevelopmental problems including internalizing and externalizing symptoms.

For example, three large prospective longitudinal studies revealed that children of mothers who were overweight or obese before pregnancy were more likely to show internalizing symptoms or negative emotionality at preschool age (Rodriguez, 2010), and throughout childhood and adolescence (Robinson et al., 2013; Van Lieshout et al., 2013a). These studies also controlled for some potential confounding or mediating variables (e.g., maternal education, stressful life events in pregnancy etc.), which did not attenuate the significance of the findings. However, after adjustment for similar confounders, data from two large European cohort studies (Brion et al., 2011) and from an Australian cohort (Van Lieshout et al., 2013b) failed to reveal a significant association of maternal overweight prior to pregnancy with maternal reports on internalizing symptoms in children aged 2 and 3 years, respectively.

Although there has been some evidence from longitudinal studies that elevated maternal BMI-scores or maternal overweight/obesity are also linked to increased levels of externalizing problems in children (Brion et al., 2011 using data derived from the Generation R cohort; Van Lieshout et al., 2013a,b), these associations have not been confirmed by other studies (Brion et al., 2011 using data derived from the ALSPAC cohort; Antoniou et al., 2014).

Van Lieshout and colleagues (Van Lieshout et al., 2011; Van Lieshout, 2013) noted that most of the studies they reviewed had some methodical flaws, such as depending on self-reported anthropometric data, which in some cases was utilized retrospectively, or not examining confounding variables. Likewise, the pathways through which maternal weight could influence children's emotional and behavioral problems are not clear, i.e., whether an intrauterine mechanism or other confounding or postnatal risk factors are responsible for this association (Brion et al., 2011; Van Lieshout et al., 2011).

In this context, problems in parenting may serve as a mediator between maternal obesity and child behavior problems (Van Lieshout et al., 2013a). There is consistent agreement that low quality parent–child interactions (e.g., characterized by harshness, hostility, intrusiveness) contribute to the development and maintenance of child psychopathologies: less warmth, abusive parenting, parental rejection but also over-involvement within the parent–child relationship, all appear to be risk factors for internalizing problems in children (McLeod et al., 2007; Yap and Jorm, 2015). Similarly, inflexible and negative behavioral patterns such as rigidity and inappropriate limit setting have been shown to be associated with higher levels of externalizing symptoms (Hollenstein et al., 2004; Middleton et al., 2009). By contrast, aspects characterizing higher emotional availability (EA) between mother and child (i.e., greater maternal structuring, non-intrusiveness, absence of hostility, and children's involvement of the mother) prior to kindergarten entry predicted lower levels of internalizing or externalizing symptoms in children 1 year later (Biringen et al., 2005). Hence, functional mother–child interactions may be a protective factor against maladaptive psychosocial development in the child within the context of maternal obesity.

Additionally, maternal parenting stress may represent another moderating or mediating risk factor through which maternal weight status may contribute to children's psychological difficulties. There is a large amount of research demonstrating that—in addition to normal parental difficulties and life events—specific parenting stress is related to children's internalizing behaviors (Anthony et al., 2005; Ashford et al., 2008; Rodriguez, 2011) and externalizing behaviors (Anthony et al., 2005; Kwon, 2007; Mackler et al., 2015). Again, these links may be established by various mechanisms such as genetic risk factors (Franic et al., 2010; Posthuma and Polderman, 2013) and biological pathways, e.g., intrauterinary exposure of the fetus to hormones involved in the stress response (Glover et al., 2010), but also via parenting behavior towards the child.

In this context and in an extension of former research, investigating children's social competence seems relevant, because the development of social competence can be considered an important resource enabling children to adjust successfully to social contexts, ensuring academic success and psychological well-being (Ladd, 2005). The construct of social competence (Waters and Sroufe, 1983; Rose-Krasnor, 1997) includes elements such as prosocial relationships with peers, communication skills, and emotional and behavioral self-regulation (Yong et al., 2014), and is negatively associated with internalizing and externalizing symptoms (e.g., Burt et al., 2008; Bornstein et al., 2010). To our knowledge, associations between maternal weight and children's developing social competence have not been investigated yet. By contrast, longitudinal associations of children's social competence with parenting stress, as well as with the quality of the mother–child relationship, are well-established: Higher parenting stress is related to deficiencies in children's social competence and regulative skills (Anthony et al., 2005; Neece and Baker, 2008; Mathis and Bierman, 2015). Higher parental emotional support, cohesiveness, parent-child reciprocity (McDowell and Parke, 2009; Feldman and Masalha, 2010; Feldman et al., 2013), and high EA (Biringen et al., 2005; Howes and Hong, 2008) are all associated with higher social competence.

To summarize, it is not yet clear whether there are psycho-emotional mechanisms, besides genetic and biological mechanisms, that may explain the effects of maternal weight on young children's weight development. Furthermore, there is a lack of knowledge about whether children of obese mothers are at risk of developing early mental health symptoms and low social competence. As pointed out earlier, the quality of the mother–child relationship and maternal parenting stress affect children's postnatal environment during early childhood and might modify the influence of maternal obesity on children's psychosocial development.

Faced with this lack of knowledge, the first aim of our study was to evaluate whether maternal weight (indicated by BMI), the quality of the mother–child relationship (i.e., EA), and parenting stress are associated with young children's weight and psychosocial development (i.e., internalizing/externalizing symptoms and social competences). We also aimed to explore whether and how the three possible predictors mentioned above interact with each other in predicting the developmental outcome. As early childhood is a critical period for biological and psychosocial development, we recruited mothers and their children from 5 months to under 4 years in order to identify risk and protective factors before the development of obesity and problem behavior takes place as the children grow older (Baillargeon et al., 2007; Ogden et al., 2014).

Our first hypothesis was that higher maternal weight (indicated by BMI) would be associated with higher weight development (indicated by BMI–SDS^1^), higher levels of internalizing as well as externalizing symptoms and a lower social competence in young offspring. Our second and third hypotheses were that the quality of the mother–child relationship (as characterized by EA) and parenting stress, respectively, would be additional and independent predictors of the developmental outcome. In order to gain knowledge about possible risk/protective factors we further explored whether these variables could moderate or mediate the associations between maternal weight and child developmental outcomes.

## Methods

### Participants

Participants were mainly recruited via kindergartens and health care professionals in and around Leipzig, Germany (Grube et al., 2013). A sample of 208 mothers and their children was selected to participate, based on self-reported parental weight and height. In line with the study aims, we oversampled for obese mothers. We extensively evaluated the families and children at three longitudinal measurement points over 3 years. Data at *t*_1_ were collected immediately after recruitment (*n* = 208, *M*_child age_ = 24.73 months, range 5–47 months); *t*_2_ took place 11 months after *t*_1_ (*n* = 172, *M*_child age_ = 35.84 months); and *t*_3_ took place 11 months after *t*_2_ (*n* = 155, *M*_child age_ = 47.23 months). *t*_2_ and *t*_3_ retained 83% and 75% of the sample from *t*_1_, respectively. Families who dropped out of the study (typically due to moving away, lack of time, or no reaction to our contact attempts) did not differ from families who remained in the study in terms of sociodemographic variables.

For data analyses we only included mother–child dyads with complete data sets on outcome variables (*N* = 194), including children aged 5–47 months with a BMI–SDS from −1.98 to 3.52. Due to oversampling of obese mothers our sample contained 73 (37.6%) obese mothers (BMI ≥ 30) and 20 (10.3%) overweight mothers (25 ≤ BMI < 30). For the social competence outcome analyses we included only children who were 3 years and older (*n* = 118) as the instruments are only applicable in this age group. This subsample did not differ from the whole sample in terms of maternal age, education, BMI or children's sex and BMI–SDS at *t*_1_. Table 1 shows the descriptive data at *t*_1_ for the included sample.

**Table 1:** **Descriptive characteristics of the sample at ***t***_**1**_, ***N*** = 194**.

**Variables**	**Total sample at *t*_1_*M* (*SD*) or *N* (%)**
**MOTHERS' CHARACTERISTICS**	
Age in years, mean (*SD*)	31.29 (4.61)
BMI, mean (*SD*)	28.18 (8.44)
**Education**	
– No degree	1 (0.5)
– Certificate of general or secondary education^b^	77 (39.7)
– General qualification for university entrance^a^	55 (28.4)
– University degree	61 (31.4)
**CHILDREN'S CHARACTERISTICS**	
Age in months, mean (*SD*)	25.38 (11.28)
**Sex**	
– Male	95 (49.0)
– Female	99 (51.0)
BMI–SDS	0.53 (1.00)^c^
BMI-percentile	64.53 (26.88)^c^

a *This group also includes mothers with the entrance qualification for a university of applied sciences*.

b *The certificate of general education is an elementary school diploma, which is obtained on successful graduation from grade 9; the certificate of secondary education is obtained on successful graduation from grade 10*.

c *Four missing*.

### Procedures

The study was approved by the ethics committee of the Medical Faculty, University of Leipzig. Parents completed consent and demographic information forms. Mothers filled in questionnaires on their own and the child's characteristics at all points of assessment. During their visit to the laboratory at *t*_1_–*t*_3_, we assessed the anthropometric data of mothers and children. We videotaped mother–child interactions in a free-play situation at *t*_1_.

### Measures

This study was part of a larger study including various measures. The complete list of measures is described in a study protocol by Grube et al. (2013). Measures used in this study are specified below.

#### Predictor variables

##### Maternal BMI at *t*_1_

Trained study personnel assessed maternal body weight and height in a standardized way at *t*_1_^2^. Mothers were weighed on calibrated scales without shoes. For the assessment of body height we used a calibrated stadiometer; adults stood free during the assessment. We calculated the BMI (weight in kilograms divided by the square of height in meters) for mothers.

##### Parenting stress at *t*_1_

To assess mothers' parenting stress, we used the 36 short form items of the Parenting Stress Index (Abidin, 1995) from the German version of the PSI (Hofecker Fallahpour et al., 2009), describing three dimensions: Parental Distress (e.g., lack of social support, conflicts with the other parent, stressors determined by constraints in other life roles), Parent–Child Dysfunctional Interaction (e.g., lack of the feeling of reinforcement by interaction with the child, feelings of being abused or rejected by the child), and Difficult Child (e.g., parent perceives the child as difficult to manage, problems with the setting of limits in older children). Higher scores indicate a higher level of parenting stress. A PSI total score (total parenting stress) is derived from the sum of the scale scores. The internal consistency in our study was Cronbach's α = 0.89.

##### Mother-child emotional availability (EA) at *t*_1_

For 16 min, mothers interacted with their child in a free-play session with standard and age-appropriate toys during their visit to the laboratory. They were instructed to interact with their child as they normally would. We videotaped parent–child interactions and assessed EA from the videos using the EA Scales (EAS; Biringen, 2008). Two female coders (the first author and another researcher) rated the videos, after having been trained by the Biringen research group and having achieved reliability for the fourth edition of the EAS. They were blind to further information on the families participating in this study. Inter-rater reliability for the two coders was assessed for 16% of the videos (mean ICC = 0.76). The EAS include six dimensions (e.g., Biringen, 2008; Biringen and Easterbrooks, 2012):

- Adult sensitivity (high scores indicate e.g., congruent parental affect, accurate perception of and an appropriate responsiveness toward the child's signals, high flexibility, high acceptance, high ability to resolve conflicts appropriately);
- Adult structuring (high scores indicate e.g., appropriate guidance of the child's activity, high support of the child's learning and autonomy);
- Adult non-intrusiveness (high scores indicate e.g., the absence of overprotection, oversuggestiveness, overdirectiveness, or interruption of the child's activity by the adult);
- Adult non-hostility (high scores indicate the absence of overt or covert signs of hostility);
- Child responsiveness (high scores indicate a strong capacity of the child to connect emotionally to the adult in a healthy way, and to respond behaviorally and emotionally appropriately);
- Child involvement (high scores indicate a high level of interest and ability of the child to include the adult in the interaction).

For each dimension the coders rated seven components on 3-point (five components) and 7-point scales (two components). This yields a score for each dimension between 7 (non-optimal on each component) and 29 (optimal on each component). An EA composite for the adult dimensions (in this study referred to as maternal EA) and for the child dimensions (referred to as child EA) were derived by summing the corresponding scale scores, with a possible range from 28 to 116 for maternal EA and from 14 to 58 for child EA.

#### Control variable

##### Maternal education at *t*_1_

We assessed maternal education based on demographic information forms and included it as a control variable in our analyses coded as follows: 1 = no qualification, 2 = certificate of general or secondary education, 3 = general qualification for university entrance, 4 = university degree (see Table 1).

#### Outcome variables

##### Children's BMI–SDS (*t*_1_ to *t*_3_)

Trained project personnel assessed children's body weight and height in a standardized way at each point of assessment (*t*_1_, *t*_2_, *t*_3_). Children who were able to stand without help were weighed on calibrated scales without shoes. To assess body height we used a calibrated stadiometer; children stood free during the assessment. We assessed body weight and height of infants using baby scales. We calculated BMI–standard deviation scores (BMI–SDS; adjusted for age and sex; Kromeyer-Hauschild et al., 2001). The BMI–SDS (or BMI z-score) is an indicator of how many standard deviations a child's BMI is below or above the average BMI of an age- and sex-adjusted reference group (e.g., a BMI–SDS score of 1 indicates that a child's BMI is 1 standard deviation above the average score). Means and standard deviations for BMI–SDS from *t*_1_ to *t*_3_ are shown in Table 2.

**Table 2:** **Descriptive statistics (means and standard deviations, ***T***-scores) for BMI–SDS, internalizing, and externalizing symptoms (***t***_**1**_, ***t***_**2**_, ***t***_**3**_)**.

	***t***_1_			***t***_2_			***t***_3_		
	**Mean**	***SD***	***T***	**Mean**	***SD***	***T***	**Mean**	***SD***	***T***
Child BMI–SDS	0.53	1.00	55	0.49	0.87	55	0.37	0.93	54
Child internalizing symptoms	5.51	4.49	45–47	5.81	4.41	45–47	6.09	4.95	47–49
Child externalizing symptoms	9.86	6.24	46–47	9.57	6.85	46–47	8.99	6.33	44–46

##### Children's internalizing and externalizing symptoms (*t*_1_ to *t*_3_)

To assess children's internalizing and externalizing symptoms, we used the German version of the Child Behavior Checklist (CBCL 1.5–5; Achenbach and Rescorla, 2000; Arbeitsgruppe-Deutsche-Child-Behavior-Checklist, 2002). This validated and reliable questionnaire includes eight scales (Emotionally Reactive, Anxious/Depressed, Somatic Complaints, Withdrawn, Sleep Problems, Attention Problems, Aggressive Behavior, Other Problems), describing problems in children aged 18 months to 5 years^3^. Parents were asked to rate 100 items on a Likert scale (0 = not true, 1 = somewhat or sometimes true, 2 = very true or often). In this study, internal consistencies (total sample, *t*_1_ to *t*_3_) for the composite scales internalizing and externalizing problems ranged from Cronbach's α = 0.79–0.90.

For the structural equation model (SEM) analyses, children's internalizing and externalizing symptoms for each point of measurement (*t*_1_, *t*_2_, *t*_3_) were modeled as latent factors each at *t*_1_, *t*_2_, and *t*_3_ with the four scale mean scores of CBCL Emotionally Reactive, Anxious/Depressed, Somatic Complaints, and Withdrawn as indicators for internalizing symptoms, and the four mean scores of the split-half scales CBCL Attention Problems and Aggressive Behavior as indicators for externalizing symptoms using the effect coding method (Little et al., 2006). To ensure measurement invariance, we fixed the corresponding factor loadings of each indicator to be the same at each measurement occasion. The measurement models for children's internalizing and externalizing symptoms showed a good fit to the data, $\chi_{\left(51,\;\text{N}\;=\;194\right)}^{2}$ = 75.46, *p* = 0.01, CFI = 0.96, RMSEA = 0.05, and $\chi_{\left(45,\;\text{N}\;=\;194\right)}^{2}$ = 101.88, *p* < 0.001, CFI = 0.95, RMSEA = 0.08, respectively. Means and standard deviations as well as T-scores for internalizing and externalizing symptoms from *t*_1_ to *t*_3_ are shown in Table 2. For all outcomes, mean scores were in the normal range for every measurement point.

##### Children's social competence (*t*_3_)

To assess children's social competence at *t*_3_, we used the Prosocial Behavior scale (five items) of the German version of the Strengths and Difficulties Questionnaire (Goodman, 1997) and the scale Social-Emotional Competence (10 items) of the VBV 3–6 (Verhaltensbeurteilungsbogen für Vorschulkinder; Döpfner et al., 1993). The Strengths and Difficulties Questionnaire (SDQ) is a widely used questionnaire assessing the behavior of children and adolescents aged four to 16 years. All items are rated on a 3-point Likert scale (0 = not true to 2 = certainly true) and consider the child's behavior over the preceding 6 months. The German version of the SDQ is a well-validated and reliable instrument (Woerner et al., 2002). In our study, the internal consistency for Prosocial Behavior was Cronbach's α = 0.72.

The VBV 3–6 is a questionnaire for the detection of behavioral problems and social-emotional competences in children aged 3–6 and shows good to satisfactory internal consistency (Renner et al., 2004). Parents were asked to rate the frequency of aspects of the child's behavior over the last 4 weeks (0 = never, 1 = rarely, 2 = sometimes, 3 = often, and 4 = very often). In this study, the internal consistency for the scale Social-Emotional Competence was Cronbach's α = 0.71.

Children's social competence at *t*_3_ was modeled in SEM analyses as a latent factor with the four split-half means of SDQ Prosocial Behavior and of VBV 3–6 Social-Emotional Competence as indicators. The measurement model for children's social competence showed a very good fit, CFI = 1.00, RMSEA = 0.00, $\chi_{\left(2,\;\text{N}\;=\;118\right)}^{2}$ = 1.97, *p* = 0.37.

#### Statistical analyses

We used SPSS statistical software, version 20.0 (SPSS Inc.) to calculate our descriptive statistics. As our data were longitudinal (based on three measurement points) and as we assessed some outcomes (social competence) via different instruments, we used structural equation modeling (SEM) for our analyses. This allows unbiased estimates to be derived for the relations between latent constructs. Applying the full information maximum likelihood (FIML) estimation method allowed handling of missing data. For our calculations we used Mplus statistical software, version 7.1. (Muthén and Muthén, 1998–2012).

Associations between manifest predictors and the development of children's BMI–SDS as well as internalizing and externalizing symptoms were tested using latent growth curve models (LGM). LGM are a type of structural equation model using repeated measures as indicators of latent growth factors. In this study, we modeled two growth factors: an intercept and a slope. The factor loadings for the first factor, the intercept, were constrained to 1 for all points of measurement. The factor loadings of the second factor, the change parameter (slope), representing growth across *t*_1_, *t*_2_, and *t*_3_ were set to −2 at *t*_1_, −1 at *t*_2_, and 0 at *t*_3_. Using this representation of time, the intercept provides the estimated mean level at *t*_3_. Hence, we refer to the intercept as level at *t*_3_. For children's BMI–SDS development, we had to fix the variance of the change parameter (slope) to zero, due to negative variance estimation (Heywood case). With this adjustment, the model showed a very good fit to the data (see Table 3). Similarly, we fixed the variance of the change parameter (slope) to zero due to negative residual variances for internalizing and externalizing symptoms. Finally, and for the same reason, we also fixed the residual variance of the latent variable for externalizing behavior at *t*_3_ to zero. Including these adjustments, the LGMs for children's internalizing and externalizing symptoms showed a good fit to the data (see Table 3).

**Table 3:** **Means (unstandardized) and variances of the level at ***t***_**3**_ and change parameter for children's BMI–SDS and internalizing and externalizing symptoms**.

	**Level at** ***t***_3_		**Change parameter**	
	**Mean**	**Variance**	**Mean**	**Variance (fixed)**
Child BMI–SDS^a^	0.37^***^	0.58^***^	−0.09^**^	0.00
Child internalizing symptoms^b^	0.17^***^	0.01^***^	0.01	0.00
Child externalizing symptoms^c^	0.36^***^	0.06^***^	−0.03^**^	0.00

a *Model fit: $\chi_{\left(\text{3},\;N\;=\;\text{194}\right)}^{\text{2}}$ = 1.04, p = 0.79, CFI = 1.00, RMSEA = 0.00*.

b *Model fit: $\chi_{\left(\text{54},\;N\;=\;\text{194}\right)}^{\text{2}}$ = 76.53, p = 0.02, CFI = 0.97, RMSEA = 0.05*.

c *Model fit: $\chi_{\left(\text{49},\;N\;=\;\text{194}\right)}^{\text{2}}$ = 111.14, p < 0.001, CFI = 0.95, RMSEA = 0.08*.

** p < 0.01;

*** *p < 0.001. Because we used latent variables based on CBCL subscales, these numbers differ from the means of the manifest sum scores reported in Table 2*.

We regressed children's BMI–SDS, internalizing and externalizing symptoms, and social competence on the manifest predictors maternal BMI, maternal education, child age, and child sex in the first step (as children's BMI–SDS scores are already adjusted for child age and sex, we omitted these variables in analyses where BMI–SDS was the outcome), see Model 1. In order to control for the influence of children's own weight on their psychosocial development, we included children's BMI–SDS at *t*_1_, *t*_2_, and *t*_3_ as time-varying covariates in our analyses.

In the second step, we first added the three predictor variables of interest (maternal parenting stress, maternal EA, and child EA; Models 2a), and then their interactions with maternal BMI (“BMI^*^maternal parenting stress,” “BMI^*^maternal EA,” and “BMI^*^child EA”; Models 2b) in order to test for possible moderation effects. In a separate analysis (Models 3), we additionally tested whether parenting stress, maternal EA and child EA were mediators of the relationship between maternal BMI and children's internalizing and externalizing symptoms, as well as children's social competence (including child age, child sex, and maternal education as control variables). For all analyses, we centered the predictor variables on their means (using the coding of –0.5 and 0.5 for bivariate categorical variables; Kraemer and Blasey, 2004).

## Results

### Descriptive data: trajectories (*t*_1_–*t*_3_) of children's weight and internalizing and externalizing symptoms

Means and variances of the level at *t*_3_ (intercept), and change parameters for children's BMI–SDS and internalizing and externalizing symptoms are shown in Table 3.

Although the average scores for children's BMI–SDS, internalizing and externalizing symptoms were in the normal range (Table 2), we found a slight decrease over time for BMI–SDS scores, as indicated by the significantly negative change parameter (*b* = −0.09, *p* < 0.01) ending at a level of 0.37 (*p* < 0.001) at *t*_3_. The same holds true for externalizing symptoms, which also decreased over time (*b* = −0.03, *p* < 0.01). Internalizing symptoms, however, remained stable (*b* = 0.01, *p* = 0.21). As there were no significant inter-individual differences in the rate of change (as indicated by the non-significant variance in the change parameter), we were only able to use the levels at *t*_3_ (intercepts) of BMI–SDS and internalizing as well as externalizing symptoms as outcome variables in further analyses.

In the following, we present the results of our analyses according to the proposed hypotheses in separate sections for each outcome variable (BMI–SDS development, internalizing and externalizing symptoms, social competence).

### Prediction of children's BMI–SDS development

In order to test our first hypothesis that maternal weight would predict children's weight development, we conducted an LGM and regressed the level of BMI–SDS at *t*_3_ (intercept) as the outcome variable on mothers' BMI and maternal level of education at *t*_1_ as predictors.

As shown in Table 4 (Model 1), only higher maternal BMI predicted higher BMI–SDS of the child. In order to test our second and third hypotheses, that mother–child EA and/or parenting stress would predict children's weight development, we added maternal parenting stress, maternal EA, and child EA as predictors. As shown in Table 4 (Model 2a) there were no significant effects of these variables, whereas the main effect of maternal BMI on children's BMI–SDS remained significant. In order to test our exploratory research question of whether EA and/or parenting stress would moderate the association between maternal weight and children's weight development, we added the three interaction terms (“BMI^*^maternal parenting stress,” “BMI^*^EA mother,” “BMI^*^EA child,” see Model 2b) as predictors. As can be seen in Model 2b, maternal BMI remained the only significant predictor. For all three models, the fit indices indicated a very good model fit to the data (see Table 4). Due to the absence of direct effects of maternal EA, child EA, and parenting stress on children's BMI–SDS, which represents a prerequisite for a mediation effect, we did not test further whether they were mediators.

**Table 4:** **Prediction of the level at ***t***_**3**_ (intercept) of children's BMI–SDS, internalizing and externalizing symptoms, as well as social competence (standardized regression coefficients β) and model fit indices of latent growth models and latent regression analysis**.

	**Child BMI–SDS (*N* = 194)**	**Child internalizing symptoms (*N* = 194)**	**Child externalizing symptoms (*N* = 194)**	**Child social competence (*N* = 118)**
**Model 1**	***R***^2^ = 0.13^*^	***R***^2^ = **0.09**	***R***^2^ = 0.13^*^	***R***^2^ = 0.17^*^
Maternal BMI	0.37^***^	0.18^†^	0.35^***^	−0.40^***^
Maternal education	0.03	−0.14	−0.03	−0.07
Child age	−	0.14	0.06	−0.09
Child sex	−	0.02	−0.03	0.23^*^
χ^2^ (*d*f)	15.32 (7)^*^	246.43 (143)^***^	293.18 (138)^***^	20.48 (17)
CFI	0.96	0.85	0.88	0.96
RMSEA	0.08	0.06	0.08	0.04
**Model 2a**	***R***^2^ = 0.13^*^	***R***^2^ = 0.31^***^	***R***^2^ = 0.33^***^	***R***^2^ = 0.32^**^
Maternal BMI	0.36^***^	0.07	0.22^**^	−0.28^*^
Maternal education	0.03	−0.13	−0.01	−0.09
Child age	−	0.10	0.01	−0.02
Child sex	−	0.00	−0.05	0.26^**^
Maternal parenting stress	−0.03	0.49^***^	0.47^***^	−0.34^**^
Maternal EA	−0.05	0.05	−0.06	0.28^*^
Child EA	0.10	−0.15	−0.09	−0.13
χ^2^ (*d*f)	20.38 (13)	289.92 (185)^***^	357.09 (180)^***^	35.91 (26)
CFI	0.97	0.86	0.86	0.91
RMSEA	0.05	0.05	0.07	0.06
**Model 2b**	***R***^2^ = 0.14^**^	***R***^2^ = 0.33^***^	***R***^2^ = 0.34^***^	***R***^2^ = 0.40^***^
Maternal BMI	0.35^***^	0.10	0.19^*^	−0.30^*^
Maternal education	0.03	−0.13	0.00	−0.11
Child age	−	0.12	0.01	−0.04
Child sex	−	0.00	−0.04	0.24^*^
Maternal parenting stress	−0.04	0.49^***^	0.45^***^	−0.29^**^
Maternal EA	−0.05	0.08	−0.05	0.24^*^
Child EA	0.09	−0.21	−0.10	−0.06
BMI^*^maternal parenting stress	0.08	0.00	0.12	−0.07
BMI^*^EA mother	0.04	0.00	0.01	0.27^*^
BMI^*^EA child	−0.04	−0.13	0.00	−0.06
χ^2^ (*df*)	28.81 (19)	341.65 (227)^***^	399.22 (222)^***^	49.11 (35)
CFI	0.96	0.85	0.86	0.88
RMSEA	0.05	0.05	0.06	0.06

* p < 0.05.

** p < 0.01.

*** p < 0.001.

† *p < 0.06. There were no significant effects of children's BMI–SDS at t_1_, t_2_, and t_3_ on the concurrent levels of externalizing and internalizing symptoms or social competence (all p < 0.05)*.

### Prediction of children's psychosocial development

#### Children's development of internalizing and externalizing symptoms

In order to test our first hypothesis that maternal weight would predict children's internalizing and externalizing symptoms we conducted two separate LGMs and regressed the levels at *t*_3_ (intercepts) of internalizing and externalizing symptoms as outcome variables on mothers' BMI, maternal level of education and child age and child sex at *t*_1_ as predictors. As presented in Table 4 (each Model 1), higher maternal BMI predicted higher externalizing symptoms, whereas there was no effect of maternal BMI on internalizing symptoms. None of the other variables were significant predictors.

In order to test our second and third hypotheses, that mother–child EA and/or parenting stress would predict or moderate children's internalizing and externalizing symptoms, we added maternal parenting stress, maternal EA, and child EA as predictors. The main effect of maternal BMI on children's externalizing symptoms remained significant. As can be seen in Table 4 (each Model 2a), maternal parenting stress significantly predicted both internalizing and externalizing symptoms of the child, but neither maternal EA nor child EA did.

After adding the three interaction terms, the main effects of maternal BMI on externalizing symptoms and parenting stress on internalizing and externalizing symptoms remained significant. There were no significant interaction terms for internalizing or externalizing symptoms (see Table 4, each Model 2b). The fit indices for all models indicated acceptable model fits to the data (see Table 4).

**Figure 1:**
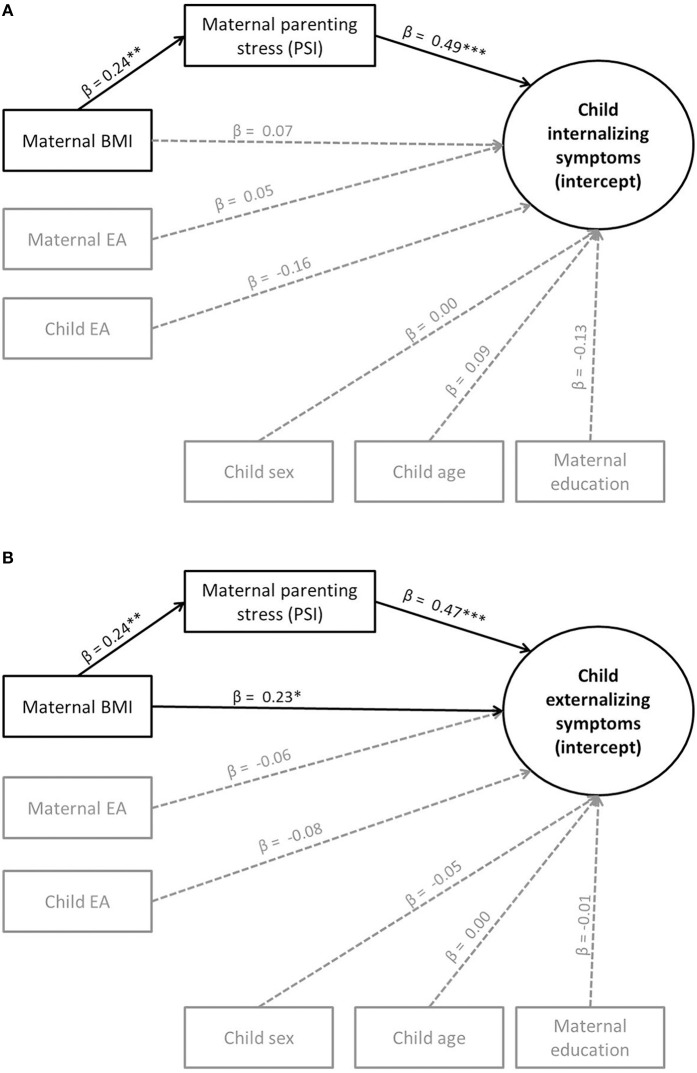
**(A)** Mediation effects (standardized regression coefficients β) on internalizing symptoms (Model 3). ^**^p < 0.01. ^***^p < 0.001. Model fit: $\chi_{\left(183,\;\text{N}\;=\;194\right)}^{2}$ = 297.58, *p* < 0.001, CFI = 0.85, RMSEA = 0.06. **(B)** Mediation effects (standardized regression coefficients β) on externalizing symptoms (Model 3). ^*^p < 0.05; ^**^p < 0.01; ^***^p < 0.001. Model fit: $\chi_{\left(177,\;\text{N}\;=\;194\right)}^{2}$ = 366.43, *p* < 0.001, and CFI = 0.86, RMSEA = 0.07.

In order to test our exploratory question of whether parenting stress would mediate the association between maternal weight and children's internalizing and externalizing symptoms, we conducted mediation analyses with children's internalizing and externalizing symptoms as outcome variables, maternal BMI as the predictor and maternal parenting stress as the mediator (including maternal EA, child EA, child age, child sex, and maternal education as control variables). Due to the absence of direct effects of maternal EA and child EA on children's internalizing or externalizing symptoms, we did not test further whether they were mediators. As shown in Figures 1A,B, maternal BMI indirectly predicted children's internalizing and externalizing symptoms via parenting stress, which therefore served as a mediator. Mothers with higher BMI showed higher parenting stress, and children of mothers with higher parenting stress showed higher maternal ratings of internalizing and externalizing symptoms. For the indirect effect from maternal BMI via parenting stress on children's internalizing symptoms (ab = 0.12), a bias-corrected bootstrap confidence interval based on 1000 bootstraps was above zero (CI: 0.04–0.19), indicating significance for this mediation (see Figure 1A). The same was true for the indirect effect (ab = 0.11) on externalizing symptoms (CI: 0.04–0.19). However, in the case of externalizing symptoms, maternal BMI continued to significantly predict children's externalizing symptoms beyond the indirect effect (see Figure 1B). The model fits for both models were acceptable (see Figures 1A,B).

#### Children's social competence at *t*_3_

In order to test our first hypothesis that maternal weight would predict children's social competence at *t*_3_, we conducted a latent regression analysis and regressed the latent factor social competence as the outcome variable on mothers' BMI, maternal level of education, child age, child sex at *t*_1_ and child BMI–SDS at *t*_3_ as predictors. As presented in Table 4 (Model 1), higher maternal BMI predicted lower social competence. Furthermore, child sex was a significant predictor, with girls showing higher social competence.

**Figure 2:**
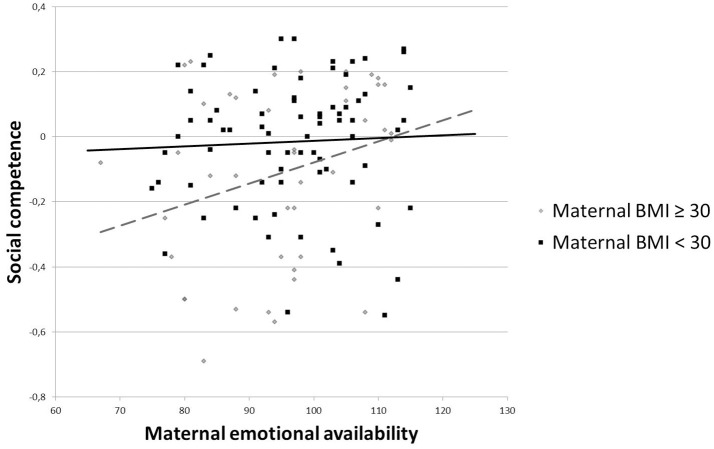
**Moderation effect of maternal EA on the relationship between maternal BMI and children's social competence (Model 2b)**.

In order to test our second and third hypotheses, we added maternal parenting stress, maternal EA, and child EA as predictors. The main effects of maternal BMI and child sex on social competence remained significant. As shown in Table 4 (Model 2a), higher maternal parenting stress predicted lower social competence. Furthermore, higher maternal EA, but not child EA, predicted higher social competence of the child. After adding the three interaction terms, the main effects of maternal BMI, child sex, maternal parenting stress and maternal EA remained significant. Moreover, the interaction “BMI^*^maternal EA” yielded significance, while none of the other interaction terms did (Table 4, Model 2b). Because of the significant interaction term “BMI^*^maternal EA,” we examined the association between maternal EA and children's social competence for mothers with a BMI less than 30 (*N* = 74) and for mothers with a BMI equal to or higher than 30 (*N* = 44). As shown in Figure 2, for mothers with a BMI as from above 30, maternal EA was more positively associated with children's social competence compared with mothers with a BMI < 30. Hence, higher maternal EA buffered the negative effect of maternal BMI on children's social competence. The fit indices for all models indicated acceptable model fits to the data (see Table 4).

**Figure 3:**
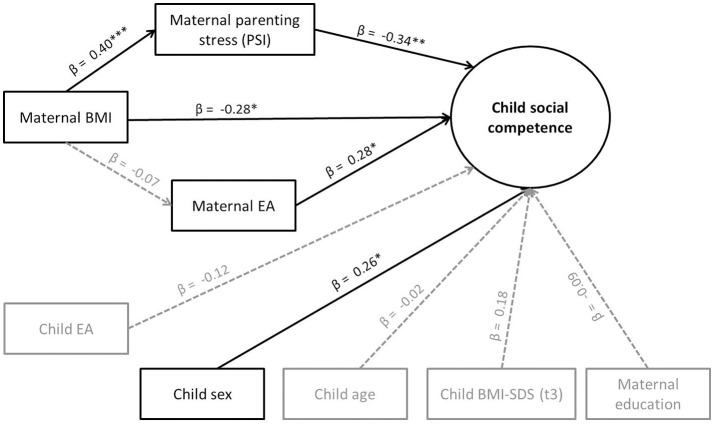
**Mediation effects (standardized regression coefficients β) on children's social competence (Model 3)**. ^*^p < 0.05; ^**^p < 0.01; ^***^p < 0.001. Model fit: $\chi_{\left(39,\;\text{N}\;=\;118\right)}^{2}$ = 53.86, *p* = 0.06, and CFI = 0.92, RMSEA = 0.06.

In order to test our exploratory question of whether maternal EA and/or parenting stress could be mediators, we conducted a mediation analysis. Due to the absence of direct effects of child EA on social competence, we did not test further whether it was a mediator. As shown in Figure 3, maternal BMI indirectly predicted social competence via parenting stress, which therefore served as a mediator. Mothers with higher BMI showed higher parenting stress, and children of mothers with higher parenting stress showed lower social competence. For the indirect effect (ab = −0.14), a bias-corrected bootstrap confidence interval based on 1000 bootstraps was below zero (−0.25 to −0.02), indicating significance. However, maternal BMI continued to significantly predict children's social competence beyond the indirect effect. There were no significant indirect effects of maternal BMI via maternal EA.

## Discussion

The results of our longitudinal study confirm the assumption that mothers' elevated BMI poses a risk to the early development of their offspring. Young children of obese or overweight mothers not only showed the development of increased weight themselves, but also showed more externalizing symptoms and lower social competence. In contrast to our prediction, maternal EA and parenting stress did not contribute to the transgenerational transmission of weight development. But these factors did play a crucial role in young children's psychosocial development: mothers' overweight/obesity was associated with more perceived stress in their parental functioning, and this higher level of stress led to more internalizing and externalizing symptoms and lower social competence of their offspring. Maternal EA, by contrast, turned out to be a protective factor and moderator: children of mothers with an elevated BMI showed lower social competence only when the mother–child interaction was characterized by low maternal EA.

### Children's weight development

In line with our first hypothesis, we found that higher maternal BMI significantly predicted higher BMI–SDS scores in children. Although on average our sample had BMI–SDS scores in the normal range, this result dovetails with previous research describing maternal BMI as a risk factor for an elevated BMI in children or the development of childhood obesity (Linabery et al., 2013; Gaillard et al., 2014).

The fact that we did not find an additional effect of mother–child EA is in contrast to studies reporting that a low quality of mother–child relationship (e.g., insecure attachment, insensitivity) increased the risk for childhood overweight or obesity (Rhee et al., 2006; Anderson and Whitaker, 2011; Wu et al., 2011; Anderson et al., 2012). Compared to our study, these studies were either cross sectional with larger cohorts, or did not control for maternal BMI/weight status (Rhee et al., 2006; Wu et al., 2011). Moreover, it might be that a possible impact of the quality of the mother–child relationship on children's weight development may become apparent only later in life (see Wu et al., 2011). We were also unable to confirm an additional effect of maternal perceived parenting stress. This result is in line with similar findings of Moens et al. (2009) and Koch et al. (2008).

The described lack of additional effects of postnatal environmental factors in our study might also be due to the fact that our sample comprised children at a very young age, in which their own development towards obesity has not yet become apparent. The effect of psychosocial factors might become more visible later as the children grow older. During these early years, the weight development of the children investigated in this study was still very stable. Alternatively, the effects of maternal weight on children's weight development may either be exclusively explained by shared genes influencing weight development (Locke et al., 2015) or by an exposure to a certain intrauterine environment accompanying maternal adiposity and increasing the risk of the child becoming obese herself, as suggested by the “fetal overnutrition hypothesis' (Lawlor et al., 2007). Moreover, other postnatal environmental factors not captured by the characteristics of maternal parenting stress or mother–child EA may play a role in children's weight development. The exposure to an obesogenic environment including e.g., the permanent availability of (energy-dense) food, a lack of physical activity, or children's unhealthy eating style (Davison and Birch, 2001), presumably add to the effect of maternal BMI.

### Children's psychosocial development

In accordance with our first hypothesis, higher maternal BMI was significantly associated with higher mother-rated externalizing symptoms. This supports the previous findings of Brion et al. (2011), and Van Lieshout et al. (2013a,b). High maternal BMI also predicted lower social competence in children. To our knowledge, this is the first study to have explored this association. Contrary to our expectations maternal BMI did not predict maternal ratings of internalizing symptoms, which again is in line with the findings of Brion et al. (2011) and Van Lieshout et al. (2013b), but contrary to those of Robinson et al. (2013), Rodriguez (2010), and Van Lieshout et al. (2013a). Hence, it remains unclear whether maternal BMI does not directly affect children's internalizing symptoms as opposed to externalizing symptoms or social competence. The non-significant findings for internalizing symptoms might be due to the young age of children in our sample. Parental reports on children at this young age may not capture internalizing symptoms as accurately as externalizing symptoms, because the latter are usually more obvious to the observer.

Our second hypothesis, that characteristics of the mother–child relationship would also predict children's psychosocial development, was only partly supported. Contrary to our hypothesis, maternal and child dimensions of EA neither directly predicted children's development of internalizing and externalizing problems nor did they serve as moderating or mediating factors, which conflicts with previous research demonstrating links between higher EA and lower levels of internalizing and externalizing symptoms (Biringen et al., 2005). This could be due to the inclusion of parenting stress as a covariate, which explained most of the variance (see below). However, higher maternal EA—but not child EA—predicted higher social competence in children. This dovetails with previous findings showing that aspects of maternal EA (particularly sensitivity and structuring) predicted later social competence in children (Biringen et al., 2005; Howes and Hong, 2008). In our exploratory analyses, maternal EA turned out to moderate the relationship between maternal BMI and children's social competence, indicating that children of mothers with an elevated BMI are at greater risk of lower social competence only when their mothers also show low levels of maternal EA. Hence, maternal EA could be regarded as a resource protecting children of mothers with an elevated BMI from social maladjustment. In consequence, EA would be a meaningful target of intervention programs with obese/overweight mothers.

In accordance with our third hypothesis, higher maternal parenting stress was strongly associated with children's internalizing and externalizing symptoms and low social competence. Moreover, high maternal BMI was associated with high maternal parenting stress, which again was related to high internalizing and externalizing symptoms and low social competence in children (mediation effects). These results illuminate a possible mechanism that seems to—at least partially—underlie the association of maternal BMI and children's psychosocial outcomes: maternal parenting stress. Previous studies included the number of stressful life events during, but not after, pregnancy (Rodriguez, 2010; Robinson et al., 2013; Van Lieshout et al., 2013a,b) or completely lacked any measures of maternal stress (Rodriguez et al., 2008; Brion et al., 2011), and none of them took into account postnatal stress arising specifically from the caregiving function. Thus, our study extends previous research and emphasizes the role of parenting stress as a relevant psychosocial factor that should be taken into account when assessing children at risk of negative psychosocial outcomes in the context of maternal obesity.

Although it was less strong, the association between maternal BMI and children's externalizing symptoms and social competence was still present even after controlling for psychosocial variables. Hence, it is up to future research to further explore mechanisms underlying these associations. Intrauterine effects that are related to maternal overweight and obesity (Anagnostis et al., 2009; Mathias et al., 2014; Ozias et al., 2015) and aspects of the obesogenic lifestyle (Hoare et al., 2014) may be worth considering in terms of the development of psychosocial problems in children.

Neither maternal education nor children's age or sex showed a significant effect on children's weight development or internalizing or externalizing symptoms. However, children's sex was significantly associated with social competence—girls were rated as being more socially competent than boys. This is in line with previous research documenting higher social competence (rated by teachers) in preschool girls compared to boys (LaFreniere and Dumas, 1996) and may be attributed to females' general advantage when it comes to decoding emotional and social cues (e.g., Hall, 1978). Moreover, children's own weight status (BMI–SDS) was not significantly associated with internalizing or externalizing symptoms or social competence. Elevated levels of behavioral and emotional difficulties as well as peer problems or social withdrawal have been reported for obese compared to non-obese children and adolescents, particularly in treatment-seeking samples (e.g., Britz et al., 2000; Vila et al., 2004), whereas in non-clinical samples these associations were less clear (Britz et al., 2000; Puder and Munsch, 2010). As our sample consisted of non-clinical participants who on average did not show internalizing or externalizing problems within the clinical range, this might explain our non-significant findings. Furthermore, the children of our sample might have been too young to have already developed fixed psychosocial patterns associated with obesity development in childhood.

## Strengths and limitations

Our study had several strengths. First, we investigated a sample of children during early childhood, a time before tendencies toward obesity and behavior problems become chronic patterns. Second, we used data from a longitudinal design, which we analyzed in a growth-modeling approach and a regression analysis using latent variables adjusted for measurement error. We also included reliable observations (rather than self-report questionnaires) of the quality of the mother–child relationship in our analyses, whereas previous research on associations between maternal and children's weight development exclusively used parental ratings and questionnaires. The inclusion of children's own weight as a control variable when examining children's psychosocial outcomes is a further strength of this study. The majority of previous studies investigating the association between maternal weight and children's psychosocial outcomes did not include children's actual weight. Besides using reliable and valid instruments of maternal parenting stress and children's psychosocial outcomes, we directly assessed anthropometric data of mothers and children in the laboratory, rather than relying on self-reported data. In comparison with direct measures of anthropometric data, self-reported weight and BMI usually tend to be underestimated, whereas height tends to be overestimated (Gorber et al., 2007), especially in case of overweight or obesity (Ciarapica et al., 2010). Hence, our results exclude this reporting bias.

Our study also had several limitations. First, our assessments of psychosocial outcomes in children relied on maternal reports alone. Some parts of the associations found, might be due to rater bias. For example, mothers with more subjective feelings of stress might also tend to rate their children as being more symptomatic. Second, our sample consisted of mainly non-clinical children with mean scores of internalizing and externalizing symptoms in the normal range. Therefore, on the one hand, our results cannot be generalized to clinical samples of children with psychiatric disorders. On the other hand, overweight or obese mothers who voluntarily participate in a study of their children's weight and psychosocial development might be more sensitive toward their children and therefore unrepresentative. This might also set limits to the generalizability of our results. Third, our study lacked assessments of children's social competence beyond one assessment point, and we had to accept a smaller sample size in this context because this measure was not suitable for children below the age of three. Fourth, regarding the ratings of EA, observers were blind to BMI scores of mothers, but not to their physical appearance. We cannot rule out that this might have affected their ratings. Fifth, from a statistical point of view, the use of linear growth models might not capture the real trajectories of children's weight development or development of internalizing or externalizing symptoms when there are other underlying forms, such as curvilinear progressions. However, such time trends are only assessable with more than three measurement occasions, which were not available in this study. The moderate to good fit of the LGMs indicates that the linear components did capture some real change. We did not investigate possible reciprocal relationships between parenting stress and children's outcomes, but as in many other studies, we conducted our research on the assumption that parenting stress increases the risk of negative outcomes in children (Tate et al., 2015). Future research should also investigate the role of parenting stress for the development of negative psychosocial outcomes in the context of maternal obesity by taking into account a possible bidirectional link between parenting stress and behavioral problems (Neece et al., 2012). Also, we assumed parenting stress to mediate the association of maternal BMI and child outcomes. However, we cannot rule out bidirectional links between BMI and parenting stress or the possibility that maternal BMI (e.g., as a result of overeating in the response to stress) at least partially mediates the association between parenting stress and child outcomes. We could not test the direction of associations explicitly in this study, however, assume that the strongest reasons (maternal BMI was rather uniform before and after birth, while parenting stress only occurred after child birth) speak for our assumed mediation between BMI and child outcomes via parenting stress. Lastly, we selected the parameters assessed in this study out of a higher number of possible parameters. This selection may influence the external validity of the results.

## Conclusions and clinical implications

To summarize, our longitudinal, non-interventional study, showed that parenting stress and mother–child EA seem to be less relevant to children's weight development, at least during early childhood. However, our study stresses the importance of taking these factors into account when addressing children's psychosocial outcomes in the context of maternal obesity. Maternal parenting stress has been identified as a potential risk factor contributing to a possibly problematic psychosocial development in terms of internalizing and externalizing symptoms and lower social competence, whereas maternal EA might represent a potential protective factor supporting children's social competence. As no conclusions in terms of causality can be drawn from our study, future interventional studies have to show whether a reduction of parenting stress and an improvement of maternal EA indeed affect children's outcomes.

It is important to emphasize that our motivation for this study was not to cause further stigmatization of obese/overweight individuals, but to ameliorate the situation of families with obesity problems. The aim of good family-oriented intervention strategies should be to help mothers to move away from debilitating feelings of guilt and toward adequate concern for their offspring's development. In this respect, our findings could be of clinical relevance if replicated and confirmed in interventional studies. Some authors have suggested intervention strategies with the primary goal of reducing maternal BMI in order to prevent adverse psychosocial outcomes in children (Robinson et al., 2013; Van Lieshout et al., 2013a). With the results of our study in mind, we would suggest adding more psychological and relational elements to the usual weight reduction programs. Thus, interventions that target the reduction of parenting stress and the improvement of the mother–child interaction quality might be helpful, particularly for psychosocial developmental outcomes in the offspring of mothers with obesity or overweight problems. Positive child outcomes usually also lead to more parental satisfaction, which could then improve the maternal psychosocial situation.

## Author contributions

SB led the writing of this manuscript and has made substantial contributions to acquisition of data, analyses, and interpretation of data. AM was involved in data analysis and writing of the manuscript and has been involved in drafting the manuscript or revising it critically for important intellectual content. VW, MG, and AKK have made substantial contributions to conception and design, acquisition of data, analysis, and interpretation of data. RG made significant contributions to data acquisition, analysis and interpretation, and revised the manuscript critically. KV was the Principal Investigator who conceived the study and led the study design and conception. AMK has made substantial contributions to conception and design of the study, data analysis, and data interpretation and has been involved in drafting the manuscript or revising it critically for important intellectual content. All authors contributed to drafting the work or revising it critically for important intellectual content, and approved the final version of the manuscript. Moreover, they all agree to be accountable for all aspects of the work.

## Funding

This work was supported by the Federal Ministry of Education and Research (BMBF), Germany, FKZ: 01EO1001.

### Conflict of interest statement

The authors declare that the research was conducted in the absence of any commercial or financial relationships that could be construed as a potential conflict of interest.

## Footnotes

^1^BMI-standard deviation scores.

^2^To control for possible intrauterine influences it would have been preferable to use mother's BMI during pregnancy. We found a very high correlation of maternal BMI at *t*_1_ with their BMI during the first two trimesters of pregnancy (*r* = 0.96, *p* < 0.001), which we obtained from mothers' records of prenatal and natal care (*N* = 171). As this was not available for all mothers, we used maternal BMI at *t*_1_ as a proxy in this study.

^3^We used the CBCL for children aged 12 months and older. Our sample also included children younger than 12 months (*N* = 23), whose scores are therefore missing but estimated by Mplus. When the analyses of this study were conducted without children younger than 12 months, the results did not change.
